# ROS production induced by BRAF inhibitor treatment rewires metabolic processes affecting cell growth of melanoma cells

**DOI:** 10.1186/s12943-017-0667-y

**Published:** 2017-06-08

**Authors:** Giulia Cesi, Geoffroy Walbrecq, Andreas Zimmer, Stephanie Kreis, Claude Haan

**Affiliations:** Life Sciences Research Unit, University of Luxembourg, 6, Ave. du Swing, L-4367 Belvaux, Luxembourg

**Keywords:** Melanoma, Metabolism, RAS/RAF/MEK/ERK pathway, NRAS, BRAF inhibitors, MEK inhibitors, Pyruvate dehydrogenase, Pyruvate dehydrogenase kinases, Reactive oxygen species, PDK inhibitors

## Abstract

**Background:**

Most melanoma patients with BRAF^V600E^ positive tumors respond well to a combination of BRAF kinase and MEK inhibitors. However, some patients are intrinsically resistant while the majority of patients eventually develop drug resistance to the treatment. For patients insufficiently responding to BRAF and MEK inhibitors, there is an ongoing need for new treatment targets. Cellular metabolism is such a promising new target line: mutant BRAF^V600E^ has been shown to affect the metabolism.

**Methods:**

Time course experiments and a series of western blots were performed in a panel of BRAF^V600E^ and BRAF^WT^/NRAS^mut^ human melanoma cells, which were incubated with BRAF and MEK1 kinase inhibitors. siRNA approaches were used to investigate the metabolic players involved. Reactive oxygen species (ROS) were measured by confocal microscopy and AZD7545, an inhibitor targeting PDKs (pyruvate dehydrogenase kinase) was tested.

**Results:**

We show that inhibition of the RAS/RAF/MEK/ERK pathway induces phosphorylation of the pyruvate dehydrogenase PDH-E1α subunit in BRAF^V600E^ and in BRAF^WT^/NRAS^mut^ harboring cells. Inhibition of BRAF, MEK1 and siRNA knock-down of ERK1/2 mediated phosphorylation of PDH. siRNA-mediated knock-down of all PDKs or the use of DCA (a pan-PDK inhibitor) abolished PDH-E1α phosphorylation. BRAF inhibitor treatment also induced the upregulation of ROS, concomitantly with the induction of PDH phosphorylation. Suppression of ROS by MitoQ suppressed PDH-E1α phosphorylation, strongly suggesting that ROS mediate the activation of PDKs. Interestingly, the inhibition of PDK1 with AZD7545 specifically suppressed growth of BRAF-mutant and BRAF inhibitor resistant melanoma cells.

**Conclusions:**

In BRAF^V600E^ and BRAF^WT^/NRAS^mut^ melanoma cells, the increased production of ROS upon inhibition of the RAS/RAF/MEK/ERK pathway, is responsible for activating PDKs, which in turn phosphorylate and inactivate PDH. As part of a possible salvage pathway, the tricarboxylic acid cycle is inhibited leading to reduced oxidative metabolism and reduced ROS levels. We show that inhibition of PDKs by AZD7545 leads to growth suppression of BRAF-mutated and -inhibitor resistant melanoma cells. Thus small molecule PDK inhibitors such as AZD7545, might be promising drugs for combination treatment in melanoma patients with activating RAS/RAF/MEK/ERK pathway mutations (50% BRAF, 25% NRAS^mut^, 11.9% NF1^mut^).

**Electronic supplementary material:**

The online version of this article (doi:10.1186/s12943-017-0667-y) contains supplementary material, which is available to authorized users.

## Background

Mutations in the Ser/Thr-kinase BRAF have been found in 10% of all human cancers with the highest prevalence observed in melanoma patients (>50%), making BRAF one of the most mutated cancer-associated genes [1, 2]. The increased kinase activity of BRAF is due to somatic point mutations, such as V600E, which result in the constitutive activation of the MAP kinase signaling pathway [3]. The mutation dysregulates cellular proliferation and confers survival advantages to cancer cells. In melanoma, activating mutations in the RAS/RAF/MEK/ERK pathway are found in many patients (50% BRAF, 25% NRAS^mut^, 11.9% NF1^mut^) while only a small part are “triple WT” and do not present one of the aforementioned hotspot mutations. Small molecule inhibitors (such as Vemurafenib also known as PLX4032 or Dabrafenib also known as GSK2118436), which bind with high affinity to the mutated form of BRAF have been successfully used as monotherapy in melanoma patients [4, 5]. However, despite the initial promising results, most patients relapse and develop drug resistance within 6 months [6]. The onset of drug resistance is often achieved by bypassing BRAF inhibition through downstream activation of MEK [7]. Therefore, a combination therapy targeting BRAF V600 mutations (Dabrafenib or Vemurafenib) as well as MEK (Trametinib or Cobimetinib) has been approved in 2015 for use in stage III and stage IV melanoma patients [8, 9]. Overall, median survival has increased with the combination therapy from 18.7 to 25.1 months [8, 9]. Thus, there is a need for novel therapies that can target melanoma efficiently as a monotherapy or delay resistance mechanisms to occur as part of a combination therapy.

Metabolic reprogramming can contribute to cancer cell survival and it is often driven by activated oncogenes or inactivated tumor suppressors such as c-Myc, HIF1α, TP53, AMPK, PI3K/AKT as well as Ras-related genes [10]. It has been shown that mutations of proteins in the RAS/RAF/MAPK pathway promote glycolysis [11] and the expression of cell surface glucose transporter 1 (GLUT1) in both colorectal cancer cell lines [12] and in melanoma [13] indicating that glucose metabolism might be important for BRAF-driven tumourigenesis. The pyruvate dehydrogenase complex (PDH) is the gatekeeper enzyme connecting glycolysis and the tricarboxylic acid (TCA) cycle. It is a multi-enzyme complex localised in the mitochondrial matrix, catalyzing the conversion of pyruvate, the end-product of the glycolysis, to acetyl coenzyme A (acetyl-CoA). Acetyl-CoA then feeds the TCA, resulting in the formation of citrate. Since the PDH is an important interface with a central role in cellular energy regulation and the supply of intermediates for many biosynthesis processes, its activity is tightly regulated. A reduction of PDH activity occurs through reversible phosphorylation of the PDH-E1α subunit on any of the three serine residues S293, S300 or S232 by kinases of the pyruvate dehydrogenase kinase (PDKs) family (PDK1, PDK2, PDK3, PDK4) [14, 15], which show a tissue-specific expression pattern and differential regulation of their activity [16, 17]. The re-activation of PDH is achieved through de-phosphorylation of the PHD-E1α subunit, catalyzed by the pyruvate dehydrogenase phosphatases (PDP1 and PDP2), which also display differences regarding their tissue distribution, regulation, and activity [16, 18]. In cancer cells, PDK inhibition and PDH activation trigger mitochondrial oxidative phosphorylation (Oxphos) and consequently ROS production which, if excessive, causes cell death. The aerobic glycolysis (also known as Warburg effect) gives the cancer cells the possibility to avoid cellular oxidative stress that would be produced by mitochondrial Oxphos for glucose metabolism.

In this study, we investigate the effects of BRAF and MEK inhibitors on metabolic proteins at early time points after treatment and analyse how BRAF-mutant cells attempt to survive under selective pressure. In particular, we demonstrate that the treatment of BRAF mutant melanoma cells with BRAF kinase inhibitors (PLX4032 or GSK2118436) increases the phosphorylation of the PDH-E1α subunit, while this is not the case in cells harboring BRAF^WT^/NRAS^mut^, a process which is facilitated by PDKs. PDH phosphorylation was also induced following inhibition of MEK1 and knock-down of Erk1/2 suggesting that downstream targets of mutant BRAF are responsible rather than off target effects of the BRAF inhibitor. In addition an activation of AMP-activated protein kinase (AMPK) was observed. Interestingly, PDH phosphorylation correlated with the appearance of an altered redox state in both mitochondria and the cytosol and was also inducible by H_2_O_2_ treatment. Finally, we show that a new PDK1 inhibitor, AZD7545, leads to efficient suppression of cell growth in cells harboring BRAF^V600E^ mutation suggesting itself as potential combination treatment with BRAF-and MEK targeting kinase inhibitors.

## Methods

### Reagents and antibodies

Selective BRAF inhibitors PLX4032 (Vemurafenib) and GSK2118436 (Dabrafenib) were purchased from Selleckchem. BRAF inhibitors were dissolved in DMSO according to the manufacturer’s instructions and stored at −80 °C. Working aliquots were diluted in 100% ethanol at a concentration of 1 mM for PLX4032 and 100 μM for GSK2118436 and stored at −20 °C. The MEK inhibitor GSK1120212 (Trametinib) was purchased from Selleckchem and was dissolved in DMSO at a concentration of 10 mM and stored at −20 °C. Dichloroacetic acid (DCA) was purchased from Sigma-Aldrich and dissolved in distilled water before use. AZD7545 was purchased from Selleckchem and was dissolved in 100% ethanol according to the manufacturer’s instructions. 10 mM working aliquots were stored at −20 °C. Mitoquinone mesylate (MitoQ) was purchased from Medkoo Biosciences, dissolved in DMSO according to the manufacturer’s instructions and stored at −20 °C. The following antibodies were used for western blot detection: anti phospho-PDH E1α (Merck Millipore); anti PDK1 (Enzo Life Sciences); anti phospho-ERK1/2 (Cell Signaling); anti ERK1/2 (Santa Cruz); anti cleaved-PARP (Cell Signaling); anti PARP (Cell Signaling); anti α-tubulin (Santa Cruz); anti HIF1α (Abcam); anti phospho- AMPK (Cell Signaling).

### Cell lines and cell culture

All melanoma cells were purchased from ATCC while 501Mel was obtained from Dr. Ruth Halaban (Dermatology department, Yale School of Medicine, USA). All cell lines were cultured in RPMI 1640 medium containing ultraglutamine (Lonza BioWhittaker), supplemented with 10% FCS (Foetal Calf Serum, GIBCO) and 1% PS (10′000 U/ml Penicillin and 10′000 U/ml Streptomycin, Lonza BioWhittaker) and grown at 37 °C in a humidified atmosphere at 5% CO2. Cells were regularly tested to be mycoplasma free. Drug-resistant cells were generated by culturing parental A375 cells in presence of 1 μΜ PLX4032 for 4–6 weeks. Twenty surviving clones were picked and then combined in equal proportions to obtain an heterogeneous pool of resistant cells.

### Plasmids

The plasmid encoding the cytosolic (cyto)-human glutaredoxin 1 (Grx1)-roGFP2 was obtained by amplifying the cDNA Grx1-roGFP2 sequence without the Peroxisomal Targeting Sequence 1 (PTS1) from the plasmid encoding the peroxisomal (po)-Grx1-roGFP2 vector [19] by PCR (primers; pCyto-EcoRI-5′ 5′-gga gga gga tca gga gga gaa ttc gtg agc aag ggc gag gag-3′ (forward) and pCyto-XbaI-3′ 5′-ctc gac tta tct aga tta ctt gta cag ctc gtc-3′ (reverse)) and subcloned into PCR2.1-TOPO (Invitrogen). The resulting plasmid was digested with EcoRI and XbaI, prior to subcloning into the EcoRI/XbaI digested pcDNA3.1-Grx1-roGFP2-PTS1 vector to obtain the p-cyto-Grx1-roGFP2. The plasmid was verified by DNA sequencing.

### Small interfering RNAs and transfection

The ERK1/2 siRNAs were obtained from GE Dharmacon (siGenome Human). siRNA transfections were performed using 3 μL HiPerfect transfection reagent (Qiagen) per reaction according to the manufacturer’s instructions. The final concentration of siRNA was 50 nM for each ERK1/2 and 100 nM for scrambled control. ERK1 and ERK2 siRNA transfections were performed 48 h prior to the 24 h incubation with PLX4032 (1 μM) and/or Trametinib (3 nM).

The PDK 1–4 siRNAs were obtained from GE Dharmacon (ON-TARGETplus Human). siRNA transfections were performed using 1.5 μL Lipofectamine RNAiMAX (Invitrogen) per reaction according to the manufacturer’s instructions. The final concentration of siRNA was 25 nM for each PDK (PDK1–4) and 100 nM for scrambled control. PDK1–4 siRNA transfections were performed 24 h prior to 24 h incubation with PLX4032 (1 μM).

### Western blot analysis and antibodies

Cell lysis was performed at 4 °C using ice cold buffers. Cells were lysed on the dish with lysis buffer containing 30 mM Tris/HCl pH 6.7, 5% glycerol, 2.5% mercaptoethanol, 1% SDS. Protein extracts were further analysed by SDS-PAGE and Western blotting. ECL signals were detected as described before [20]. Before re-probing, blots were stripped as described before [21]. All experiments were performed in three biological replicates and one representative replicate is shown. Western blot quantification of ECL signals was performed using both the Image Lab 4.0.1 software from Bio-RAD and Bio1D analysis package (Vilber).

### Quantitative PCR procedure

Total RNA was extracted using the Quick-RNA™ miniprep kit (Zymo Research) according to the manufacturer’s instructions and the concentration and quality was determined using a NanoDrop Spectrophotometer. 250 or 500 ng of total RNA was reverse-transcribed with the miScript II RT kit (Qiagen) in a volume of 10 μL, according to the manufacturer’s instructions. Quantitative real time PCR (qPCR) was carried out on a CFX96 Detection System (BioRad) in a total volume of 10 μL (10 pmol of each primer and containing cDNA corresponding to 50 ng RNA template). The housekeeping genes PPIA, HPRT and the target genes were assayed in parallel for each sample. Melting curve analysis was performed to guarantee the specificity of the qPCR primers as previously described [22]. All samples were run in biological triplicates each consisting of three technical replicates.

Gene-specific qPCR primers for PDK1, PDK3, PDK4 and HIF1a were purchased from Eurogentec (Belgium). For the detection of PDK2 a RT2 qPCR primer assays from Qiagen were used. The geometric mean of two housekeeping genes was calculated and a normalization factor for each sample was generated using geNorm (VBA add-in for Microsoft Excel). The normalization factor was used to calculate the relative amount of each target mRNA in each sample. Each sample was normalized to the untreated control.

### Detection of ROS by confocal microscopy

Cells were transfected with a plasmid coding for Grx1-roGFP2 or mitochondrial (mito)-roGFP2 (pMF1762) as previously described [23], using Lipofectamine LTX2000 (Invitrogen). Two days post transfection the cells were either subjected to a 1 h pretreatment with mitoQ (150 nM), a treatment with PLX4032 (3 h for A375; 10 h for 501 Mel or IGR37) or a combination thereof. roGFP2 is a genetically engineered GFP, which contains two cysteine residues that can be oxidized. Depending on the oxidation state, roGFP changes its excitation spectrum (reduced: 488/530 nm; oxidized: 400/530 nm). By measuring the ratio (400/488 nm) of the fluorescence emitted by the two excitation states of the roGFP2, the redox status of a subcellular compartment can be monitored [24, 25]. The higher this ratio, the higher is the redox state. The analysis was performed using the ImageJ software on pictures taken using an Andor Revolution W1 spinning disc confocal microscope, mounted on a Nikon Ti microscope (60× oil objective). Between 10 and 20 ROIs (regions of interest) were analysed per cell with a minimum of 12–20 cells analysed for each condition. The excitation record time was set to 250 msec or 300 msec for the 400 nm channel and 50 msec or 60 msec for the 488 nm channel for the Grx1-roGFP2 or mito-roGFP2 respectively. Two independent experiments were performed.

### Real-time proliferation assays

50 × 10^3^ cells/well of 5 melanoma cell lines were seeded in 12-well plates and 24 h later stimulated with 10 μM of AZD7545. Cellular growth was monitored in the IncuCyte ZOOM live cell microscope (Essen BioScience) and images were taken in phase contrast every 3 h for a total of 90 h. Proliferation assay were carried out for three biological replicates and for each figure one representative replicate is shown.

### Preparation of the A375-iRFP cell line

A375 cells were plated in 12 well plates in complete medium and allowed to adhere overnight. Cells were transduced with lentiviral particles (LV-iRFP-P2A-Puro) containing near-infrared fluorescent protein (iRFP) linked to the puromycin resistance gene (Puro) via a P2A cleavage peptide (Imanis Life Sciences). Virus was prepared in serum-free medium and added to the cells with a multiplicity of infection (MOI) of 10. To increase transduction efficiency, the plate was centrifuged at 300 g for 30 min and placed in the incubator. Four hours after transduction, complete medium was added to the cells. Forty-eight hours after transduction, cells were checked on the Odyssey Infrared imaging system (LI-COR Bioscience) for iRFP expression and selection with puromycin (Invivogen) at 1 μg/ml was initiated. After several rounds of puromycin selection, cells were analysed on the Odyssey Infrared imaging system (LI-COR Bioscience) and on a FACSCanto II flow cytometer using FACSDiva (BD Bioscences) software to confirm that 100% of the cell population is expressing iRFP.

### Long term proliferation assay

The A375-iRFP cells were used to test the combination of 1 μM PLX4032 and 10 μM AZD7545 versus PLX4032 alone. 10,000 cells were seeded per well (6 well plates) and were treated with the inhibitors for 3 weeks. Medium was changed twice a week. After 3 weeks of treatment, cells were scanned and the intensity of the iRFP signal was measured using the LI-COR Odyssey instrument (LI-COR Biosciences). The iRFP signal was quantified using the Image Studio lite version 4.0 software (LI-COR Biosciences).

### 3D spheroid growth assay

A375 cells were plated in round-bottom ultra-low attachment 96-well plates in 90 μl of serum-free DMEM-F12 supplemented with B-27 (1X; Invitrogen), Insulin (4 U/L; Sigma), Heparin (4 μg/ml; Sigma), EGF (20 ng/ml; Biomol); bFGF (20 ng/ml: Miltenyi Biotec), and penicillin/streptomycin (1X, Lonza). After 2 days of sphere formation, drugs were added. Sphere growth was monitored in the IncuCyte ZOOM live cell microscope (Essen BioScience) by measuring four different spheroid diameters after 3 days. Spheroid assays were carried out in three biological replicates.

## Results

### BRAF inhibitors lead to an increase of PDH phosphorylation in BRAF mutant (V600E) but not in BRAF wild-type melanoma cells

**Fig. 1:**
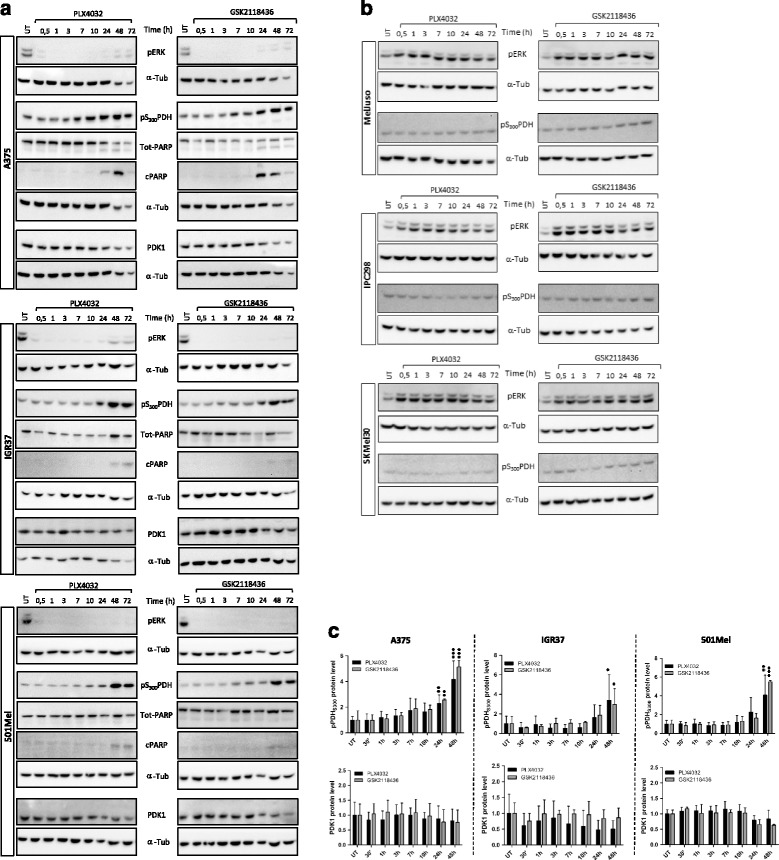
BRAF inhibitors induce upregulation of PDH-E1α phosphorylation in BRAF^V600E^ melanoma cells but not in wild-type BRAF melanoma cells. Western blot analysis of A375, IGR37 and 501 Mel cells (BRAF^V600E^) (**a**) and MelJuso, IPC298 and SKMel30 cells (BRAF^WT^/NRAS^mut^) (**b**) treated with 1 μM of PLX4032 and 100 nM of GSK2118432 for the indicated time points. α-Tubulin was used as loading control; representative blots of three biological replicates are shown. **c** Quantification of pPDH and PDK1 levels for A375, IGR37 and 501Mel, normalized to the untreated control. Error *bars* represent the standard deviation of three biological replicates. Statistical significance was determined using one-way ANOVA coupled with Dunnett’s multiple comparisons tests. *p > 0.05, **p > 0.01, ***p > 0.001

In the wake of recent publications investigating the role of the BRAF oncogene on metabolic processes, we wanted to explore the effects of BRAF inhibitors on melanoma cells. To this end, time course experiments were performed in a panel of BRAF^V600E^ and BRAF^WT^/NRAS^mut^ human melanoma cells, which were incubated with two BRAF kinase inhibitors, PLX4032 and GSK2118436, for the indicated time points (Fig. 1). As expected, BRAF inhibition caused the down-regulation of phosphorylated ERK in the BRAF^V600E^ positive cell lines A375, IGR37 and 501Mel already after 30 min (Fig. 1a). On the contrary and as previously described [26, 27], PLX4032 and GSK2118436 lead to an upregulation of ERK phosphorylation in cells expressing BRAF^WT^/NRAS^mut^ (Fig. 1b). Total PARP and cleaved PARP detections were performed to assess the induction of apoptosis which seems to occur only 48 and 72 h after treatment of the BRAF^V600E^ positive cells. Interestingly, we found an increased phosphorylation of PDH-E1α on S_300_ from around 7 h post inhibitor treatment in the BRAF^V600E^ positive cell lines A375, IGR37 and 501 Mel (Fig. 1a and c), increasing at 24 h and generally peaking at 48 h. In the BRAF^WT^/NRAS^mut^ cells, the situation was again different and no change in the phosphorylation of PDH was observed over time. Phosphorylation of PDH-E1α at S_300_ is associated with an inhibition of the activity of the pyruvate dehydrogenase complex (PDH), which converts pyruvate into acetyl-CoA and thus regulates the entry of metabolites from glycolysis into the TCA cycle. Our data suggest that treatment with BRAF inhibitors induces changes in the metabolism of cells harboring the BRAF^V600E^ mutation in a different way than in cell expressing BRAF^WT^.

### PDH phosphorylation is mediated by Erk

**Fig. 2:**
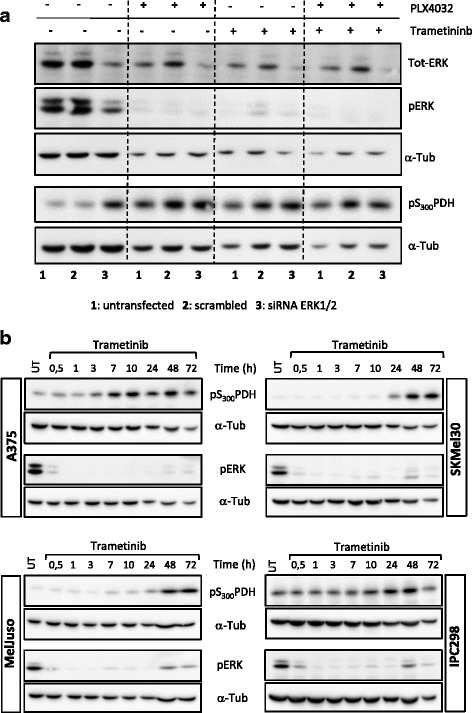
Knock-down of ERK1/2 by siRNAs and treatment with a MEK1 inhibitor induces pS_300_PDH. **a** A375 cells were transfected with siRNA against ERK1/2 (50 nM each siRNA) or a scrambled control (100 nM) for 72 h. 48 h prior to collection, the cells were incubated with either PLX4032 (1 μM) or Trametinib (3 nM) or both drugs. One representative of three biological replicates is shown. **b** Western blot analysis of A375 (BRAF^V600E^), MelJuso, IPC298 and SKMel30 cells (NRAS^mut^) treated with 5 nM of Trametinib for the indicated time points. α-Tubulin was used as loading control; representative blots of three biological replicates are shown. 1: *Untransfected*; 2: *scrambled*; 3: *ERK1/2 siRNA*

To rule out that the PDH phosphorylation is an off target effect of the BRAF inhibitor and since BRAF^V600E^are known to activate ERK1/2 via MEK1, we investigated the involvement of ERK1/2 and MEK1 in mediating PDH-E1α phosphorylation in A375 cells. Using an siRNA approach targeting both ERK1 and ERK2 in combination with PLX4032 we could show that a knock-down of ERK1/2 induces an upregulation of PDH-E1α phosphorylation, comparable to what was achieved with PLX4032 (Fig. 2a). In addition, the same upregulation was obtained when the cells were treated with a MEK1 inhibitor (Trametinib), which also leads to the down-regulation of pERK. Of note, neither of the effects could be increased by combination treatment with both BRAF and MEK inhibitor. To further explore the link between RAS/RAF/MEK/ERK pathway and PDH phosphorylation we used NRAS^mut^ cells (SKMel30, MelJuso, IPC298) (which did not upregulate pPDH upon BRAF inhibitor treatment) and treated them with Trametinib for the indicated time points. As expected, MEK inhibition caused a down-regulation of ERK phosphorylation already after 30 min (Fig. 2b) also in NRAS^mut^ cells. Phosphorylation of PDH-E1α on S_300_ was observed in those cells from around 24 h onwards. These results suggest that the phosphorylation of PDH is a specific effect mediated by the BRAF/MEK/ERK pathway and not an off target effect of BRAF inhibitors and that it occurs in all melanoma cells presenting activating mutations in this pathway.

### Pyruvate dehydrogenase kinase (PDK) mRNA levels are upregulated upon BRAF inhibitor treatment

**Fig. 3:**
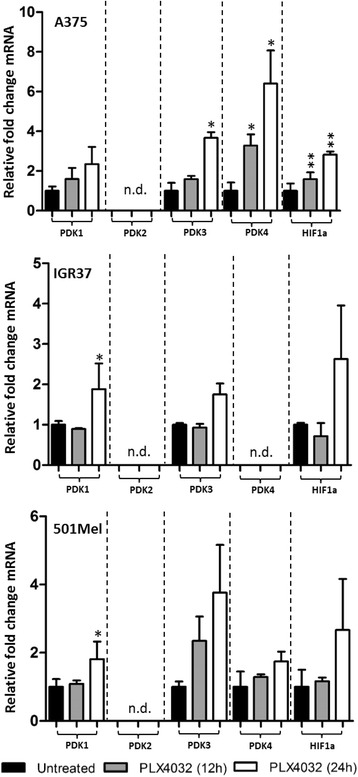
PLX4032 treatment induces upregulation of PDK mRNA in BRAF^V600E^ melanoma cells. Quantitative RT-PCR of PDKs and HIF-1α mRNA. The fold change was calculated relative to untreated controls. Error *bars* represent the standard deviation of three biological replicates. PDK2 was not detectable in *tested cell lines* (Cq ≥ 30) while PDK4 (Cq ≥ 30) was not detectable in IGR37 cells only. Error *bars* represent the standard deviation of three biological replicates. Statistical significance was determined in comparison to the untreated control using paired Student’s *t*-tests. *p > 0.05, **p > 0.01, ***p > 0.001

Since PDKs are known to be responsible for PDH phosphorylation [14, 15], we next analysed mRNA levels of the four PDK isoforms in three BRAF^V600E^ cell lines. HIF1α was included in the study since this transcription factor induces PDK1 expression and has been shown to be upregulated by conditions other than hypoxia [28], notably also by activation of the RAS/MAPK pathway [29]. Furthermore, constitutive HIF1α activity has been described in malignant melanoma [30]. Surprisingly, an upregulation of the mRNA levels of PDK1 and HIF1α was detected in all cell lines at 12 and 24 h after PLX4032 treatment (Fig. 3). However, validation of these results showed that although HIF1α mRNA was upregulated upon treatment, this effect was not detectable at the protein level (Additional file 1: Figure S1). This finding was corroborated by the fact that PDK1 protein levels, which are regulated by HIF1α protein, were also not upregulated upon BRAF inhibitor treatment. Interestingly, the mRNAs of the other PDK family members PDK3 and PDK4 were upregulated upon BRAF inhibitor treatment, while PDK2 was not detected in any cell line. The protein levels of PDK3 and PDK4 could not be investigated since the available antibodies were not specific for these proteins (data not shown). Thus, mRNA levels of the PDKs are upregulated following BRAF inhibitor treatment, although for PDK1 no increase in protein levels was detectable.

### PDKs are crucial for the observed PDH-E1α phosphorylation

**Fig. 4:**
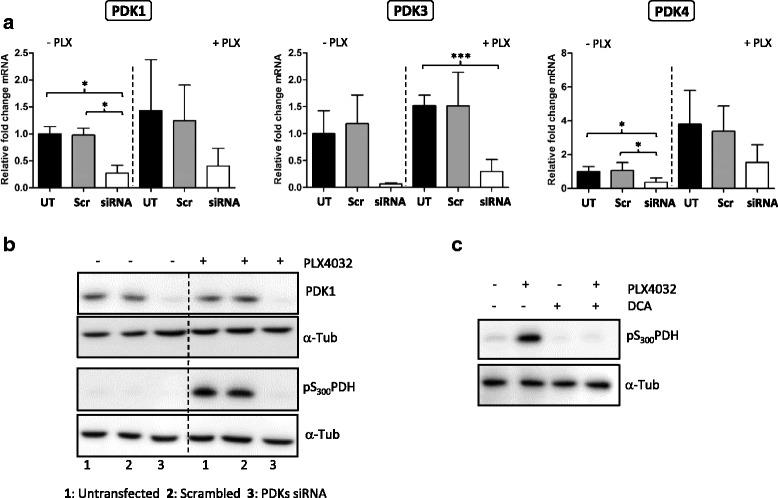
PDKs are crucial for PDH phosphorylation. A375 cells were transfected with siRNA against all four PDK isoforms (PDK1–4; 25 nM siRNA for each isoform) or 100 nM of scrambled control; after 24 h cells were incubated with PLX4032 (1 μM) for another 24 h. **a** qPCR analysis of PDK1–4 mRNA levels to show the efficiency of the siRNA knock-down of each siRNA and (**b**) corresponding western blot analysis. **c** Western blot analysis of A375 treated with PLX4032 (1 μM for 24 h), DCA (20 mM for 48 h) or both drugs. Error *bars* represent the standard deviation of three biological replicates. For each western blot experiment, one representative of three biological replicates is shown. Statistical significance was determined using paired Student’s *t*-tests. *p > 0.05, **p > 0.01, ***p > 0.001. 1: *Untransfected*; 2: *scrambled*; 3: *PDKs siRNA*

The activity of PDKs can also be regulated by alternative post-translational mechanisms [31], and for enzymes the regulation of their activity is often more important than their mere expression level. Therefore, we investigated the importance of PDKs in our inhibitor-mediated PDH-E1α phosphorylation by knocking down all four PDK isoforms using an siRNA approach. The down-regulation of mRNAs for all isoforms ranged between ~10–40% compared to both untreated and scrambled controls (Fig. 4a). As for PDK1 protein levels, a strong reduction following the siRNA treatment could be observed too (Fig. 4b). Following the knock-down of the PDKs, a drastic decrease in the phosphorylation levels of PDH-E1α at serine 300 residue in A375 cells was detected, despite the presence of PLX4032 (Fig. 4b). To further support these results, we used dichloroacetic acid (DCA), a pan-PDK inhibitor, which also resulted in a decreased phosphorylation level of PDH-E1α in A375 (Fig. 4c). In summary, these data indicate that PDK isoforms mediate the PLX4032/GSK118436-induced phosphorylation of PDH-E1α.

### BRAF inhibitors also trigger the phosphorylation of AMP-activated protein kinase (AMPK)

**Fig. 5:**
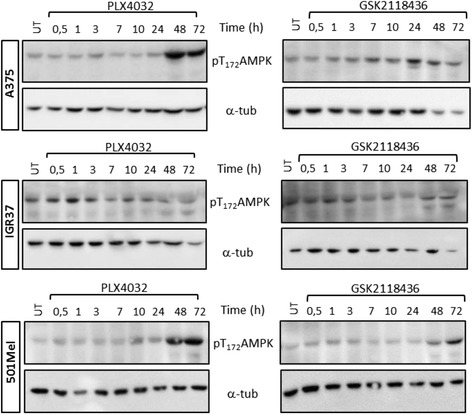
PLX4032 and GSK2118436 induce upregulation of AMPK phosphorylation in BRAF^V600E^ melanoma cells. Western blot analysis of A375, IGR37 and 501 Mel cells (BRAF^V600E^) treated with 1 μM of PLX4032 and 100 nM of GSK2118432 for the indicated time points. α-Tubulin was used as loading control, and one representative blot is shown

AMPK phosphorylation and its subsequent activation upon BRAF inhibitor treatment has previously been described in melanoma and colorectal cancer cells [32, 33]. Since AMPK has indirectly been implicated in mediating PDH phosphorylation [34], we explored the phosphorylation status of AMPK in our setting. In accordance with previous publications [32, 33], we also observed an upregulation of AMPK phosphorylation on T_172_ following inhibition of BRAF^V600E^ with both PLX4032 and GSK2118436 (Fig. 5). However, the phosphorylation occurred generally at later time points (24 or 48 h after treatment) compared to the onset of PDH phosphorylation. For this reason, we exclude the possibility that the observed PDH phosphorylation following BRAF inhibitor treatment was indirectly induced by AMPK.

### Reactive oxygen species (ROS) are upregulated upon PLX4032 treatment and correlate with PDH phosphorylation

**Fig. 6:**
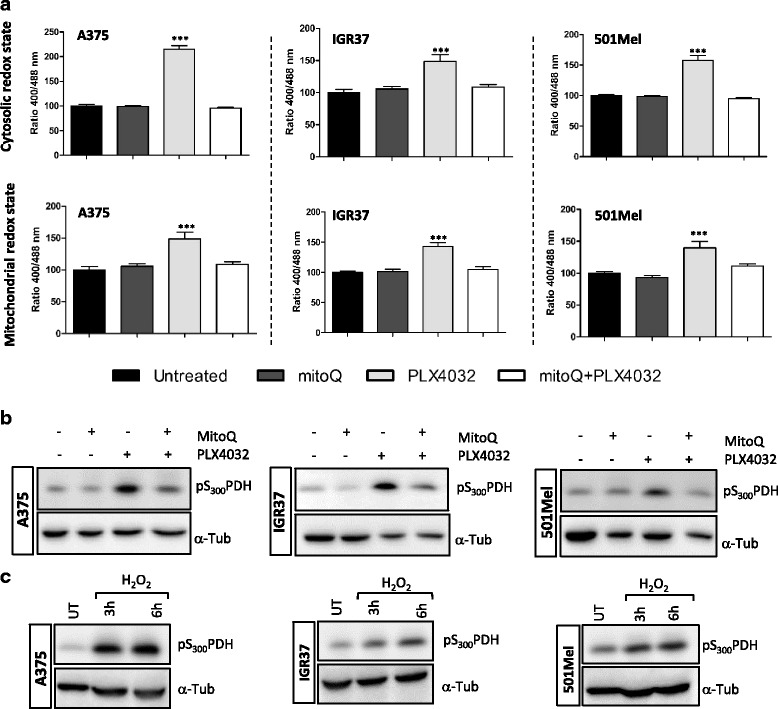
ROS are induced after PLX4032 treatment of melanoma cells. **a** A375, 501 Mel or IGR37 cells were transiently transfected with plasmids encoding Grx1-roGFP2 or mito-roGFP2. Two days post transfection the cells were either subjected to a 1 h pretreatment with mitoQ (150 nM), a treatment with PLX4032 (3 h for A375; 10 h for 501Mel or (IGR37) or a combination thereof. Following this, the redox status was measured in the cytosol or mitochondria. Data were normalized to untreated cells, followed by one-way ANOVA coupled with Dunnett’s multiple comparisons tests. Representative graphs of two biological replicates are shown. **b** Western blot analysis of A375, IGR37 and 501Mel pre-treated for 1 h with mitoQ (150 nM) followed by 24 h of 1 μM PLX4032 treatment. Results are shown for one representative of three biological replicates. **c** Western blot analysis of A375, IGR37 and 501 Mel treated with hydrogen peroxide (H_2_O_2_; 100 μM) for the indicated time points. Results are shown for one representative of three biological replicates. *p > 0.05, **p > 0.01, ***p > 0.001

Mitochondrial ROS production after PLX4032 exposure has recently been described [35]. Since we and others have previously demonstrated that the induction of ROS can stimulate the phosphorylation of PDH [34, 36], we investigated a possible connection between ROS and pPDH after PLX4032 treatment in the context of melanoma. In order to measure the redox state in both mitochondria and the cytosol after PLX4032 treatment, A375, IGR37 and 501Mel cells were transfected with a plasmid expressing either the mito-roGFP2 or the Grx1-roGFP2, which can be used to monitor the mitochondrial and the cytosolic redox state, respectively. Both redox states after PLX4032 treatment were compared to untreated controls. In particular, we treated A375 cells with the BRAF inhibitor for 3 h whereas IGR37 and 501Mel were tested for 10 h. The time points were chosen according to the onset of pS_300_PDH after PLX4032 treatment, in particular just before the phosphorylation was becoming prominent. Furthermore, we also used a mitochondria-targeted antioxidant (mitoQ) in order to prevent or decrease oxidative stess induced by PLX4032. Melanoma cells were pre-treated with 150 nM of the antioxidant, mitoquinone mesylate (MitoQ), followed by PLX4032 treatment. For all cell lines, both the mitochondrial and the cytosolic redox status was significantly increased after exposure to PLX4032 and decreased in case of mitoQ pre-treatment (Fig. 6a). We could also observe a decreased phosphorylation of PDH-E1α on S_300_ when the cells were pre-incubated with MitoQ suggesting that ROS are activating PDKs, which in turn leads to inhibition of PDH by phosphorylation (Fig. 6b). As an additional confirmation, cells were treated with 100 μM H_2_O_2_, which also induced an upregulation of PDH-E1α phosphorylation (Fig. 6c).

### AZD7545 (PDK inhibitor) leads to efficient growth suppression in cells harboring BRAF and NRAS mutations as well as in inhibitor-resistant melanoma

**Fig. 7:**
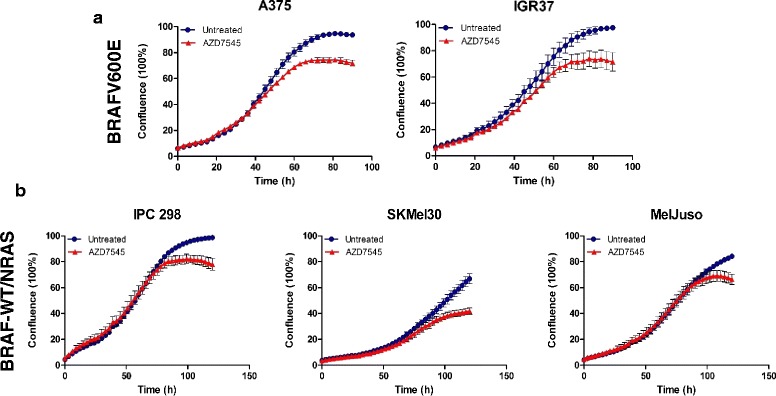
AZD7545 mediated growth suppression of BRAF^V600E^ mutant and NRAS^mut^ melanoma cells. Twenty-four hours after plating, both A375 and IGR37 *cell lines* (BRAF^V600E^) (**a**) and SKMel30, IPC298 and MelJuso *cell lines* (NRAS^mut^) (**b**) were treated with 10 μM of AZD7545. The plates were imaged using an IncuCyte ZOOM live cell microscope (Essen BioScience) and images were taken every 3 h for a total of 90 h (BRAF^V600E^) and 120 h (NRAS^mut^). Results are shown for one representative of three biological replicates

**Fig. 8:**
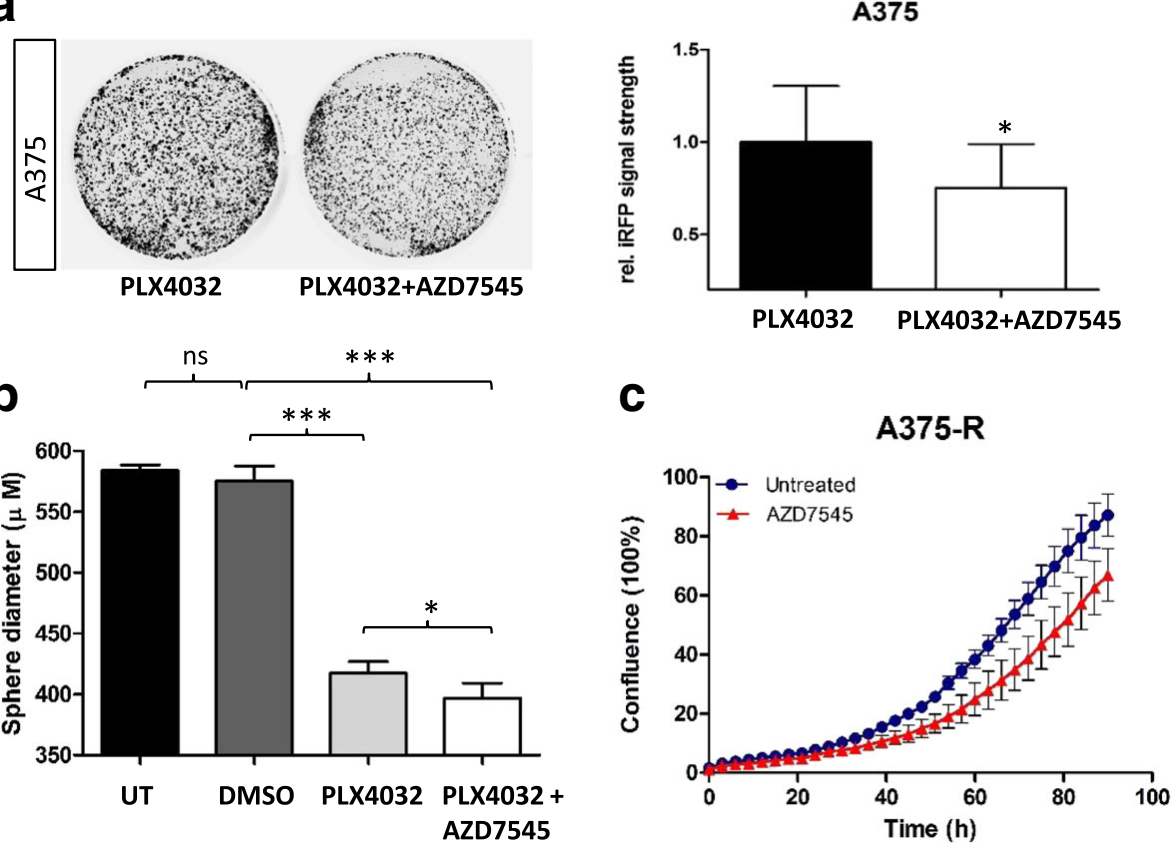
Combination of AZD7545 and PLX4032 more efficiently suppresses melanoma growth compared to each compound alone. **a** Represenative experiment of A375 melanoma cells expressing iRFP treated either with 1 μM of PLX4032 or with 1 μM of PLX4032 in combination with 10 μM AZD7545 for 3 weeks. The intensity of red fluorescence was quantified and the bar diagram represents three biological replicates with their standard deviation. **b** Spheroid cultures of A375 melanoma cells were treated with DMSO control, with 1 μM of PLX4032 or with 1 μM of PLX4032 in combination with 10 μM AZD7545. After 3 days sphere diameters were measured and represented as bar diagrams. Error *bars* represent the standard deviation of a minimum of four technical replicates of one representative experiment of three biological replicates. **c** Twenty-four hours after plating, BRAFi-resistant A375 melanoma cell (A375-R) were stimulated with 10 μM of AZD7545. The plates were imaged using an IncuCyte ZOOM live cell microscope (Essen BioScience) and images were taken every 3 h for a total of 90 h. Results are shown for one representative of three biological replicates. Statistical significance was determined using paired Student’s *t*-tests. *p > 0.05, **p > 0.01, ***p > 0.001

Generally a metabolic shift from glycolysis to glucose oxidation has been associated with an increase in the electron transport chain activity and ROS production [37] and oxidative stress in form of increased ROS levels drives cells into apoptosis [38]. Based on this, we hypothesize that the inhibition of PDKs during acute BRAF inhibitor treatment and during early development of resistance might be beneficial for patients as such that sufficient levels of ROS could be generated, which might drive cancer cells into apoptosis and thus delay or prevent resistance. In this context, it has previsouly been shown that the PDK inhibitor DCA negatively affects the growth of melanoma cells, which are sensitive or resistant to BRAF inhibition by suppressing glycolysis [39]. First we tested a PDK inhibitor with low micromolar cellular potency, AZD7545, as single treatment on both BRAF^V600E^ and BRAF^WT^/NRAS^mut^ human melanoma cells. Interestingly, we detected a 30–40% decrease in cell growth in BRAF^V600E^ A375 and IGR37 cell lines (Fig. 7a). Interestingly the AZD7545-mediated suppression of growth could also be observed in BRAF/NRAS^mut^ cells, although, at later time points. This might be attributed to the slower growth of those cell lines (Fig. 7b), which also signal via the MAPK pathway. These data suggest that AZD7545, which we used at 10 μM, might be a valid alternative to Dichloroacetate (DCA), another PDK inhibitor used at millimolar concentrations. To investigate if AZD7545 could delay or influence the occurrence of BRAF inhibitor resistance we tested the combination of the two inhibitors on melanoma cell growth. A375 melanoma cells were transduced with lentiviral particles containing iRFP and were treated for 3 weeks either with 1 μM of PLX4032 or with 1 μM of PLX4032 in combination with 10 μM AZD7545. By measuring the intensity of the iRFP signal, we observed that cells treated with both inhibitors showed a more pronounced reduction in cellular growth (Fig. 8a). This drug combination was also tested on sphere models with similar results (Fig. 8b). It is tempting to speculate that the targeting of PDKs in combination with BRAF inhibitors could be a promising strategy to more efficiently hit melanoma cells or to delay the onset of drug resistance. In addition BRAFi-resistant A375 melanoma cells also showed reduction of growth upon AZD7445 treatment (Fig. 8c).

## Discussion

Metabolic reprogramming, often driven by activated oncogenes, is a well known feature of cancer cells. Recent studies have shown a link between oncogenic BRAF signaling and metabolic reprogramming in melanoma (for a comprehensive review see [40]), making the targeting of metabolic pathways a potentially interesting therapeutic strategy. Melanoma has been described to be highly glycolytic, due to upreguation of glucose transporters and lactate dehydrogenase-A (LDH-A) [41–43]. Inhibition of BRAF^V600E^ suppresses GLUT1/3 and Hexokinase 2 protein levels leading to reduced levels of lactate and ATP, thus showing that BRAF inhibition counteract the Warburg effect [13]. In the present study, we demonstrate that administration of BRAF inhibitors induces phosphorylation of proteins involved in the cellular metabolism, notably via PDH. Furthermore, inhibition of MEK1 alone or in combination with BRAF inhibitors as well as siRNA knock-down of ERK1/2 also mediated phosphorylation of PDH, indicating that it is not an off target effect of the BRAF inhibitor but an effect mediated by the RAS/RAF/MEK/ERK pathway (Figs. 1, 2a and b). PDH is the key enzyme linking glycolysis to the TCA. PDH-E1α phosphorylation at the serine residues 293, 300, and 232 is known to be responsible for the down-regulation of its activity. Thereby, conversion of pyruvate to acetyl-CoA is prevented, which contributes to a down-regulation of the TCA and Oxphos.

We demonstrate that PDKs are required for the observed effect since siRNA-mediated knock-down of the PDKs clearly impairs the inhibitor-mediated upregulation of phosphorylation. Administration of the PDK inhibitor DCA had the same effect. Interestingly, the expressed PDK1, PDK3 and PDK4 proteins are regulated either by HIF-mediated transcription (PDK1), increasing ATP levels (PDK3) or amino acid starvation (PDK4). Here, we did not observe changes in HIF1α or PDK1 protein levels. ATP has been described to be decreased upon BRAF inhibition [13] and it is thus unlikely to account for PDK2 activation. In our short term 2D cell culture (the effect is seen from 7 h post inhibitor treatment) experiment, nutrient deprivation can hardly be responsible for PDK4 activation [44]. Thus alternative activation mechanisms have to be taken into account. Protein kinases can be redox-sensitive [45] and can either be activated or inactivated by ROS. We have recently shown that PDKs were activated by ROS in the first hours of hypoxic conditions [36] and that PDH phosphorylation can be mediated by ROS-dependent activation of PDKs [34, 36]. In the current study, we detected an increased mitochondrial and cytosolic redox state upon administration of PLX4032 in BRAF-mutant positive melanoma cells and we also observed that exogenously applied H_2_O_2_ was sufficient to increase PDH phosphorylation in melanoma cells, indicating that ROS might indeed be responsible for the observed effect. In the presence of a mitochondria-targeted ROS scavenger, MitoQ, the BRAF inhibitor failed to induce PDH phosphorylation, which strongly points to an activation of PDKs by ROS.

Cancer cells produce higher levels of ROS compared to nontransformed cells, due to metabolic reprogramming or to changing fluxes of metabolites or to nutrient and oxygen fluctuations in the tumour microenvironment. Interestingly, any disturbance of the steady state of the electron transport chain (ETC.) seems to generate ROS [46, 47]. As one example, increasing and decreasing oxygen levels both induce the production of more ROS by the ETC. In the same way, activation of BRAF by mutation in melanoma should lead to the induction of genes involved in antioxidant defence, since MAPK activation was described to induce such a response [48]. As a consequence, inhibition of BRAF might increase ROS levels by suppressing the transcription of these antioxidant genes. In addition NRF2, a transcription factor regulating antioxidant defence, is known to be activated by ERK, other MAPKs and Akt. Thus, BRAF mutations also activate antioxidant defence downstream of the NRF2 pathway. Again, BRAF inhibitor treatment might also lead to increases in ROS by this mechanism.

**Fig. 9:**
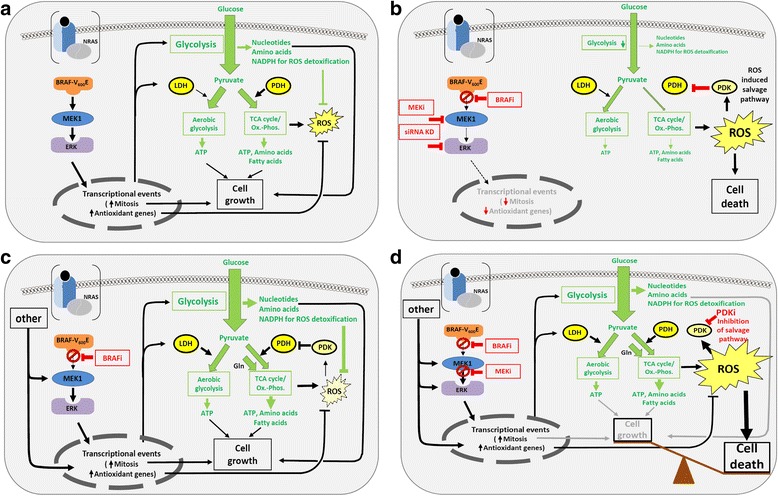
Model summarising metabolic changes in sentitive versus resistant melanoma cells upon BRAF inhibition. **a** BRAF^V600E^, in addition to its well known effects on the cell cycle and anti-apoptosis, leads to upregulation of glucose uptake and LDH-A, promoting aerobic glycolysis and cell growth. Moreover, activation of BRAF, leads to the induction of genes involved in antioxidant defense. **b** Upon treatment with BRAF inibitors, or other MEK/ERK pathway inhibitors, glycolysis is inhibited. Changes in metabolic fluxes through the TCA cycle together with the inhibition of the transcription of antioxidant genes, induce ROS. The increasing levels of ROS activate PDKs, which in turn inactivate PDH, thus reducing pyruvate use in the TCA cycle, which in a negative regulatory loop, inhibit further ROS production. **c** In BRAF inhibitor resistance, the MAPK pathway is reactivated by compensatory mechanisms and as a consequence glycolysis and antioxidant-defence genes are reactivated. Glycolysis produces ATP, nucleotides (via the pentose phosphate pathway (PPP)), NADPH (for antioxidant defense via PPP) and amino acids. The cells become addicted to an oxidative metabolism with glutamine (Gln) feeding anaplerotically into the TCA cycle to produce NADH, amino acids, ATP (via Oxphos), fatty acids (via AcetylCoA) and ROS. These ROS can be kept in check by NADPH, which is produced by the PPP and by antioxidant gene transcription downstream of the RAS/RAF/MEK/ERK pathway. At need, PDKs are activated by ROS and PDH is inhibited. This prevents pyruvate from entering the TCA and ROS to reach toxic levels. **d** In BRAF inhibitor resistance of melanoma, pharmacological inhibition of PDKs releases the brake on pyruvate entry into the TCA and causes unchecked pyruvate use, associated with an increase in ROS production. This increase in ROS tilts the balance towards cell death. Thus potent nonomolar PDK inhibitors could efficiently reduce the viability of BRAFi-resistant cells or might prevent or delay BRAFi resistance

Melanoma cells have been discussed to have a highly oxidative metabolism and thus treatment with BRAF or MEK inhibitors, increase oxidative stress within the cancer cell by upregulating ROS (as we show here). The increased phosphorylation of PDH by PDKs that we describe can be interpreted as a protective response of the cancer cells to prevent further ROS production. The oxidative activation of PDKs, due to increasing ROS levels, prevent pyruvate from entering the TCA and the ETC. (which is the major source of ROS) and stop ROS from reaching toxic levels. PDK inhibitors thus release the block on pyruvate entry into the TCA and consequently lead to an increase in ROS levels that can be toxic to cells and contribute to cancer cell death (Fig. 9). BRAF inhibitor treatment inhibits the TCA cycle and contributes to the decrease in ATP production, which was recently reported by several studies [13, 49] and by our preliminary measurements (data not shown). Interestingly, we observed that in PLX4032-resistant melanoma cells the PDH phosphorylation upregulation was overcome (Additional file 2: Fig. S2) suggesting a metabolic adaptive process occurring during drug treatment (Fig. 9).

This ROS-mediated regulation of kinases is an interesting emerging field in cancer cell biology. It seems that ROS can either activate or inactivate kinases by oxidation. Interestingly, ROS regulate some kinases directly involved in metabolic processes, which are also involved in regulation of ROS itself. One such mechanism we now describe in this paper: The ROS-mediated activation of PDKs lead to phosphorylation of PDH, which in turn limits influx of pyruvate into the TCA and the Oxphos (or the ETC.). This represents a negative regulatory loop limiting the generation of more ROS. In the same way, ROS inhibit PKM2 (a cancer-expressed isoform of pyruvate kinase), leading to accumulation of glycolytic intermediates, which feed into the pentose phosphate pathway to generate NADPH. NADPH in turn plays an essential role in ROS detoxification [50]. Along the same lines, ROS have been described to activate AMPK, a kinase regulating mitophagy/autophagy, which can be interpreted as a pathway restricting further ROS production [51, 52].

Interestingly, the BRAF^V600E^ oncogene signaling regulates PDH phosphorylation in very different ways in Oncogene Induced Senescence (OIS) [53] and in BRAF inhibitor resistance (this paper and [13]), which occur in different steps of oncogenesis or treatment (see also Fig. 9). In a melanoma mouse model, OIS in BRAF^V600E^ positive cells was mediated by down-regulation of the TCA and Oxphos. In this system, PDH down-regulation by phosphorylation was mediated by PDK1 down-regulation and PDP2 upregulation at the protein level. Loss of OIS, characteristic of progression to cancer leads to a reversal of these effects [53].

DCA has been tested in multiple cell culture and rodent models of cancer, and PDK1 knock-down has been described to enhance the sensitivity of BRAF^V600E^ positive melanoma to BRAF inhibitors. We tested a pan-PDK inhibitor, AZD7545, which interferes with the lypoyl binding pocket of PDKs for its capacity to inhibit the growth of BRAF^V600E^ and NRAS^mut^ positive cells. We observed that AZD7545 suppressed growth of BRAF^V600E^ positive cells and kinase inhibitor-resistant cells when applied in μM concentrations. Interestingly, AZD7545 had no effect on keratinocytes (HaCaT) and normal fibroblasts, cell types which constitute the cutaneous microenvironment of melanoma tumors (data not shown), indicating selective effects on BRAF/NRAS-mutated or resistant cancer cells. Finally, we show that the combination of BRAF inhibitors with PDK inhibitors is more efficient in tumor growth suppression than the single treatment suggesting that the simultaneus targeting of metabolic pathways might indeed be beneficial for melanoma patients.

## Conclusions

Current guidelines for advanced stage melanoma foresee the use of targeted therapy followed by immunotherapy if kinase inhibitor treatments are not effective or once patients become resistant. Appropriate first line treatments are selected based on specific features of the patient, including BRAF mutation status. For BRAF-mutated melanoma patients, targeted combination therapy with BRAF and MEK inhibitors is recommended, a treatment line, which has been approved in 2015. Until then, BRAF inhibitors were given alone but combined BRAF and MEK inhibition was shown to improve the overall survival [54]. Despite these encouraging results, double drug resistance is likely to occur in some patients [55]. Triple target therapy approaches are currently under investigation, especially those targeting pathways other than the RAS/RAF/MEK/ERK. Furthermore, about 50% of patients do not carry the BRAF mutation and not all of those might be suitable candidates for immunotherapy. Finally, intrinsic resistance exists in a group of melanoma patients carrying BRAF mutations but not responding to BRAF and MEK inhibitors. For this large group of melanoma patients, alternative therapeutic targets need to be identified. A deeper understanding of metabolic changes under selective pressure may contribute to the identification of such novel target lines.

In this study, we report that RAS/RAF/MEK/ERK pathway inhibition induces increased ROS levels, which activate PDKs. PDKs then phosphorylate and thereby inhibit PDH, which reduces further ROS production by the TCA cycle. Consequently, PDK inhibitors should increase ROS production significantly by preventing blockade of pyruvate entry into the TCA cycle. The use of a specific PDK inhibitor combined with BRAF inhibition or with BRAF and MEK inhibitors might thus increase ROS production to levels, which initiate cell death and in this way delay or prevent BRAF inhibitor resistance. Given the early onset of resistance to BRAF inhibitors in virtually all treated patients together with the still limited success rates of immune therapy [56], there is a pressing need for efficient therapies in late stage melanoma patients, which either prevent or considerably delay drug resistance by targeting other aberrant signaling pathways. More potent PDK inhibitors with nanomolar cellular potency targeting the lipoyl- or the ATP-binding pockets of the kinase domains, might be a promising inhibitor to add in combination with BRAF and/orMEK kinase inhibitors.

## Additional files

Additional file 1: Figure S1. PLX4032 and GSK2118436 do not induce up-regulation of HIF-1α protein in BRAFV600E melanoma cells. Western blot analysis of A375, IGR37 and 501Mel cells (BRAFV600E) treated with 1 μM of PLX4032 and 100 nM of GSK2118432 for the indicated time points. HIF-1α protein was not detectable in all three cell lines. Positive control: A375 short term hypoxia. (PDF 155 kb)

Additional file 2: Figure S2. BRAF inhibitors do not induce phosphorylation of PDH in resistant melanoma cells. Western blot analysis of untreated A375, A375 cells resistant to Vemurafenib (A375-R) and under constant presence of 1 μM of PLX4032 and A375 cells stimulated with 1 μM of PLX4032 for 24 h. α-Tubulin was used as loading control; representative blots of three biological replicates are shown. (PDF 102 kb)

## Abbreviations

AcetylCoA: Acetyl coenzyme A
AMPK: AMP-ctivated Protein Kinase
BRAF: B-Rapidly accelerated fibrosarcoma
DCA: Dichloroacetic acid
ERK: Extracellular signal regulated kinase
LDH-A: Lactate dehydrogenase-A, Gln, glutamine
mTOR: Mechanistic target of rapamycin
Oxphos: Oxydative phosphorylation
PDC: Pyruvate dehydrogenase complex
PDH: Pyruvate dehydrogenase
PDKs: Pyruvate dehydrogenase kinases
PDP1 and PDP2: Pyruvate dehydrogenase phosphatases
ROS: Reactive oxygen species
TCA: Tricarboxylic acid cycle
