# Supplementary figures and images for: ROS production induced by BRAF inhibitor treatment rewires metabolic processes affecting cell growth of melanoma cells
Source: Mol Cancer. 2017 Jun 8;16:102. doi: 10.1186/s12943-017-0667-y (PMC5465587; doi:10.1186/s12943-017-0667-y)

Figure S1

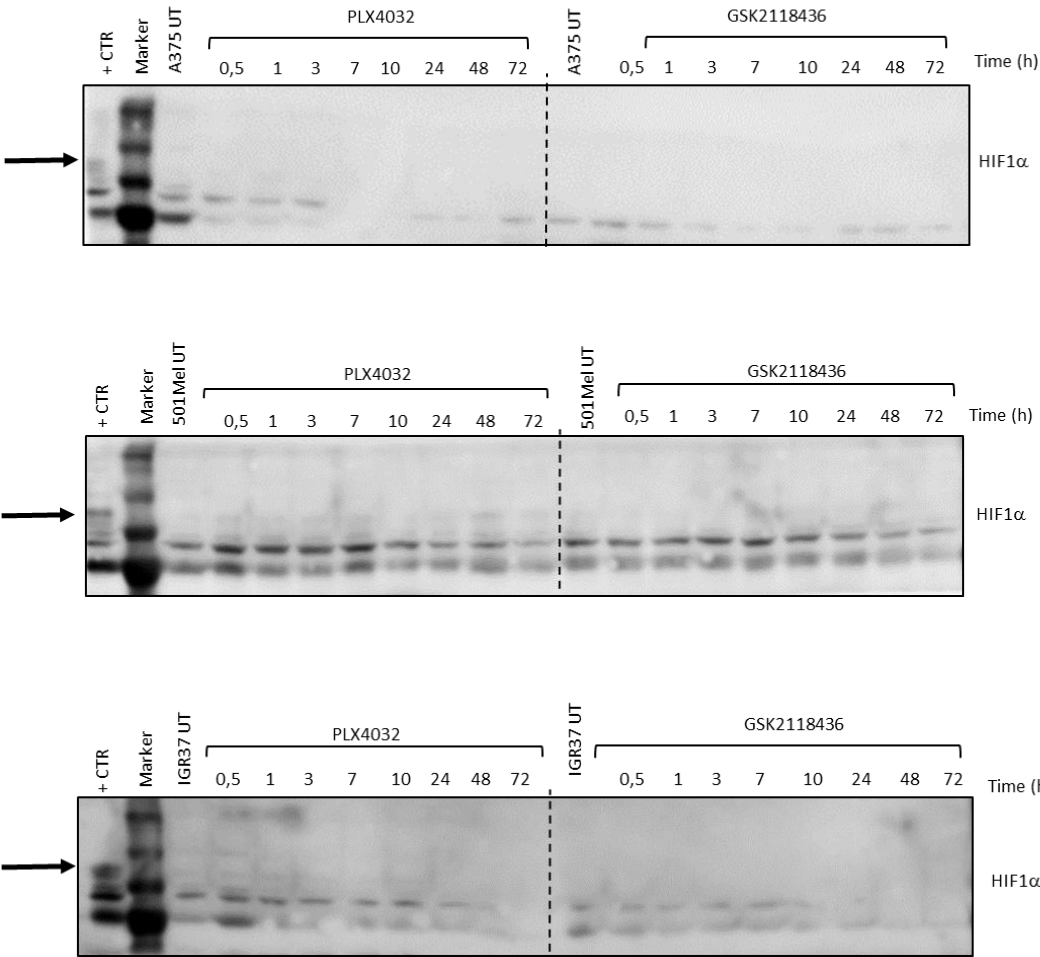

Supplement: Supplementary file 1 — PLX4032 and GSK2118436 do not induce up-regulation of HIF-1α protein in BRAFV600E melanoma cells. Western blot analysis of A375, IGR37 and 501Mel cells (BRAFV600E) treated with 1 μM of PLX4032 and 100 nM of GSK2118432 for the indicated time points. HIF-1α protein was not detectable in all three cell lines. Positive control: A375 short term hypoxia. (PDF 155 kb) [file 12943_2017_667_MOESM1_ESM.pdf]

Figure S2

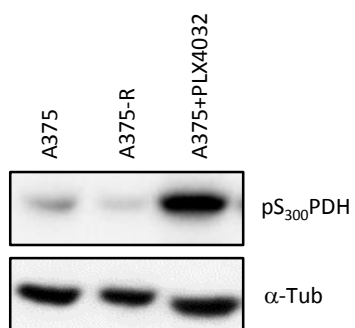

Supplement: Supplementary file 2 — BRAF inhibitors do not induce phosphorylation of PDH in resistant melanoma cells. Western blot analysis of untreated A375, A375 cells resistant to Vemurafenib (A375-R) and under constant presence of 1 μM of PLX4032 and A375 cells stimulated with 1 μM of PLX4032 for 24 h. α-Tubulin was used as loading control; representative blots of three biological replicates are shown. (PDF 102 kb) [file 12943_2017_667_MOESM2_ESM.pdf]
